# Prognostic impact of late gadolinium enhancement cardiac magnetic resonance in the risk stratification of heart transplant patients

**DOI:** 10.1186/1532-429X-18-S1-O63

**Published:** 2016-01-27

**Authors:** Patrizia Pedrotti, Claudia Vittori, Rita Facchetti, Stefano Pedretti, Santo Dellegrottaglie, Angela Milazzo, Maria Frigerio, Cristina Giannattasio, Alberto Roghi, Ornella Rimoldi

**Affiliations:** 1 IBFM, CNR, Segrate, Italy; 2 Cardiology, Ospedale Niguarda, Milan, Italy; 3 Health Science Dept, Bicocca University, Milan, Italy; 4 Clinica Villa dei fiori, Acerra, Italy

## Background

The aim of the present study was to assess the association of the presence and amount of late gadolinium enhancement (LGE) at cardiac magnetic resonance (CMR) with cardiovascular adverse events in patients who survived the first year after cardiac transplantation (HTx).

## Methods

We enrolled 48 patients (mean age, 54.7 ± 14.6 years; 37 men) at various stages after HTx (median 9.88 yrs). All patients underwent standard CMR at 1.5 T, images were acquired with retrospective ECG gating and during repeated single breath-holds. Balanced steady-state free precession were used to obtain cine images (echo time/repetition time, 1.6/3.2 ms; flip angle, 60°; pixel size, 2.4 × 1.4 mm, slice thickness 8 mm, gap 2 mm) in three long-axis planes and in contiguous short-axis slices. LGE images were acquired in matching short-axis and long axis planes starting 10 min after intravenous injection of gadolinium-DTPA (Schering^©^, Berlin, Germany; 0.15 mmol/kg) and using a segmented inversion-recovery gradient echo sequence. The primary endpoint was a composite of first occurrence of major cardiovascular adverse events which required hospitalization (MACE): CV death, congestive heart failure, redo transplant, arrhythmias requiring hospitalization (high degree A-V block, sustained supraventricular tachyarrhythmias, sustained ventricular tachycardia, bradycardia requiring pace-maker implantation), coronary revascularization, the secondary endpoints were all-cause death and cardiovascular death. Survival of patients was estimated by Kaplan-Meier analysis, for each end point Cox multivariate models were constructed.

## Results

Myocardial LGE was detected in 26 patients (54%) with a median amount of 9.16 g (IQR 3.01 to 21.21) corresponding to 6.4% (IQR 2. 3 to 13.25) of LV mass. Cardiac allograft vasculopathy (CAV) was diagnosed in 54% LGE positive and 23% LGE negative patients (p = 0.057). Primary and secondary end-points were recorded during the follow-up period median 5.16 years (IQR 4.33 to 6.52). Ten patients died and 26 were readmitted because of MACE. LGE presence at CMR was associated with an increased risk of sustaining an index event during follow up shown by the Kaplan-Meier survival curves (Figure, logrank test p = 0.003). Multivariate Cox analysis identified as independent predictors of MACE a diagnosis of CAV (HR 3.63;p = 0.0039), left ventricular end systolic volume index (HR1.04; p = 0.008), LGE mass (HR1.04;p = 0.0007), LGE % of left ventricular mass (HR1.083; p = 0.0002). Independent predictors of all-cause death were CAV (HR 6.33;p = 0.0201), LGE mass (HR1.04; p = 0.005), LGE % of left ventricular mass (HR1.075; p = 0.007) whereas cardiovascular death was predicted by CAV (HR 10.8; p = 0.0263) and LGE mass (HR1.04; p = 0.0037).

## Conclusions

In survivors after 1 year from HTx the presence of CAV and the amount of LGE are strongly associated with MACE and mortality.

**Figure 1:**
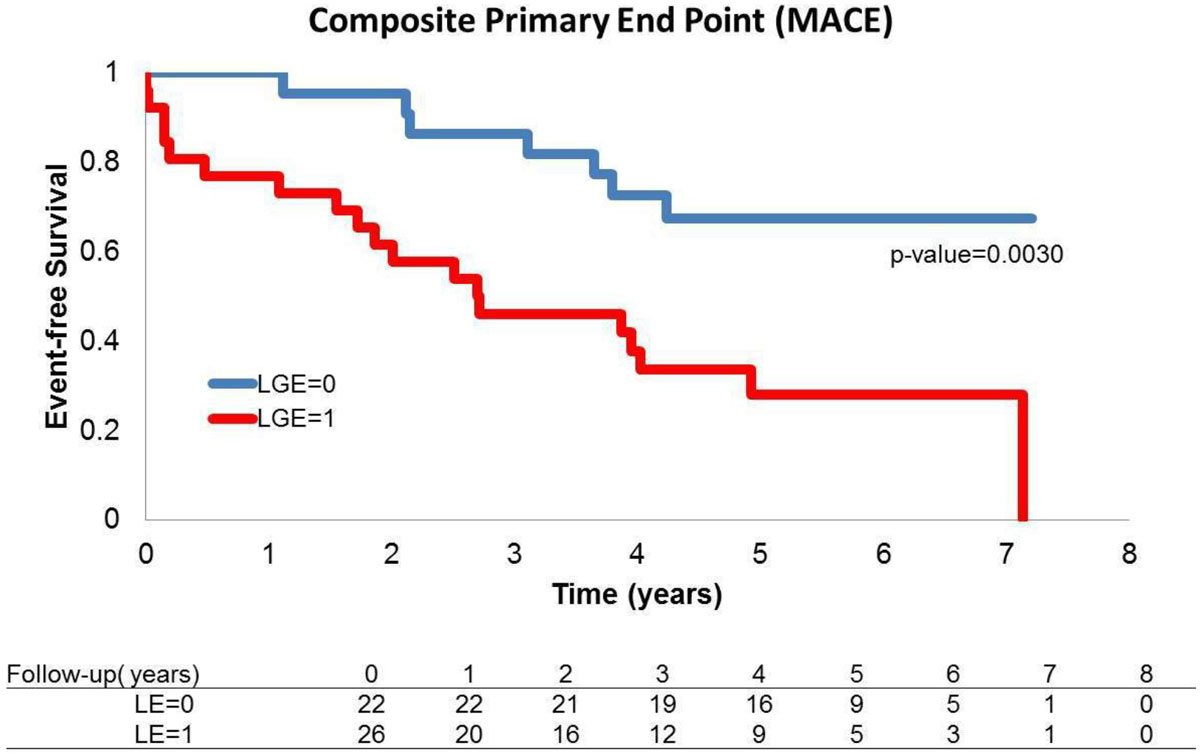
**Kaplan-Meier event-free survival curve for occurrence of MACE patients grouped by presence or absence of LGE**.

